# Supplementary material for: Diet Quality and Risk of Parkinson’s Disease: The Rotterdam Study
Source: Nutrients. 2021 Nov 7;13(11):3970. doi: 10.3390/nu13113970 (PMC8618850; doi:10.3390/nu13113970)
Supplement: Supplementary file 1 [file nutrients-13-03970-s001.zip › nutrients-1417911-supplementary.pdf]

Supplementary Tables: Diet Quality and risk of Parkinson's disease: the Rotterdam study

Table S1. Description of the food groups.

Food Group	Included food Products
Vegetables	Raw and boiled vegetables, pickled vegetables, raw salads, mushrooms, onions and garlic
Fruits	Fresh fruits
Legumes	Beans, lentils and legume soup
Milk and cream	All-fat-milk, chocolate milk, coffee creamer and cream
Yoghurt and fermented milk	All-fat-yoghurt, buttermilk, fruit-yoghurt and quark
Eggs	Boiled eggs and baked eggs
Cheese	Cheese and cheese spread
Unprocessed white meat	Chicken
Processed and red meat	Pork, beef, minced beef, horse meat, lamb, hamburger, sausages, ham, liver and bacon
Fish and seafood	Herring, white fish, salmon, seafood, sardines, tuna and fish fingers
Vegetable oils and spreads	Peanut oil, margarine, soy-oil, half-fat margarine, safflower-oil and olive-oil
Animal-based fats	Dairy butter, frying fat, baking fat, lard and other fats
Wholegrain products	Wheat germs, brown bread, wheat bread, oat flakes, muesli, brown rice, spelt, wholegrain pasta, wholegrain cereals and porridge with wholegrains.
Refined grain products	Cornflakes, currant-bread, gingerbread, Dutch rusk, white bread, pancakes, white rice, pasta, crackers and croissants
Potatoes and fries	Cooked potatoes, mashed potatoes, fries and oven fries
Sugar-containing beverages	Soda, diet soda, all fruit juices, lemonade, alocohol free beer and vegetable juices
Tea	Tea
Coffee	Coffee
Alcoholic beverages	Wine, beer, gin, sherry and other liquors
Savory snacks	Salted biscuits, crispy nuts and other salty snacks
Sweet snacks	Pies, cakes, biscuits, chocolate, sugar, acid drops, licorice and candy bars
Nuts and seeds	Linseeds, unsalted nuts, salted peanuts, other seeds
Soy products and meat replacers	Tofu, tempeh, soy chunks, soy drink, soy yoghurt, meat replacers

Table S2: Time-stratified analyses for association between dietary patterns and Parkinson's disease.

	Total No. of Incident Parkinson's Disease	Basic Model HR (95% CI)	Covariate Model HR (95% CI)
Dutch diet quality score			
Follow-up 0–5 years	40	0.99 (0.70–1.40)	0.98 (0.68–1.39)
Follow-up 0–10 years	78	0.93 (0.73–1.18)	0.92 (0.72–1.17)
Follow-up 0–15 years	101	0.95 (0.77–1.17)	0.92 (0.75–1.14)
Mediterranean diet score			
Follow-up 0–5 years	40	1.07 (0.75–1.53)	1.06 (0.73–1.54)
Follow-up 0-–10 years	78	0.97 (0.76–1.23)	0.96 (0.75–1.22)
Follow-up 0–15 years	101	1.00 (0.81–1.23)	0.97 (0.78–1.20)
Prudent pattern			
Follow-up 0–5 years	40	1.20 (0.79–1.83)	1.24 (0.79–1.93)
Follow-up 0–10 years	78	0.98 (0.72–1.35)	1.00 (0.71–1.42)
Follow-up 0–15 years	101	0.94 (0.71–1.25)	0.92 (0.67–1.26)
Unhealthy pattern			
Follow-up 0–5 years	40	1.01 (0.68–1.51)	1.05 (0.69–1.61)
Follow-up 0–10 years	78	0.99 (0.76–1.29)	1.02 (0.77–1.35)
Follow-up 0–15 years	101	0.95 (0.76–1.20)	1.01 (0.79–1.29)
Traditional Dutch pattern			
Follow-up 0–5 years	40	0.89 (0.61–1.29)	0.82 (0.50–1.36)
Follow-up 0–10 years	78	0.89 (0.69–1.15)	0.84 (0.60–1.19)
Follow-up 0–15 years	101	0.91 (0.73–1.14)	0.83 (0.62–1.13)

The hazard ratios (HR) and 95% confidence interval (CI), obtained using Cox Proportional Hazard models, are shown per standard deviation (SD) for the dietary patterns and scores. The basic model was adjusted for sex, age at baseline and Rotterdam Study cohort. The covariate model was adjusted for all items in the basic model and additionally for body mass index (BMI), education, smoking behavior and energy intake.

Table S3: Analyses per cohort for association between dietary patterns and Parkinson's disease.

	No. of Incident Parkinson's Disease/No. of Participants in Cohort	Basic Model HR (95%CI)	Covariate Model HR (95% CI)
Dutch diet quality score			
RS-I	101/5250	0.99 (0.80–1.22)	0.95 (0.77–1.18)
RS-II	16/1601	0.91 (0.51–1.61)	0.92 (0.51–1.67) [a]
RS-III	12/2563	0.91 (0.46–1.82)	0.96 (0.45–2.05) [a]
Mediterranean diet score			
RS-I	101/5250	0.99 (0.80–1.24)	0.94 (0.75–1.18)
RS-II	16/1601	0.78 (0.49–1.26)	0.79 (0.48–1.29) [a]
RS-III	12/2563	0.88 (0.49–1.59)	0.95 (0.48–1.88) [a]
Prudent pattern			
RS-I	101/5250	0.87 (0.62–1.23)	0.77 (0.53–1.12)
RS-II	16/1601	0.84 (0.41–1.71)	0.90 (0.41–1.98) [a]
RS-III	12/2563	0.90 (0.48–1.68)	1.07 (0.47–2.41) [a]
Unhealthy pattern			
RS-I	101/5250	0.93 (0.72–1.19)	0.98 (0.76–1.27)
RS-II	16/1601	1.15 (0.71–1.87)	1.20 (0.71–2.03) [a]
RS-III	12/2563	0.94 (0.51–1.73)	0.92 (0.46–1.84) [a]
Traditional Dutch pattern			
RS-I	101/5250	1.13 (0.91–1.42)	0.97 (0.71–1.32)
RS-II	16/1601	0.67 (0.37–1.23)	0.63 (0.29–1.36) [a]
RS-III	12/2563	0.70 (0.34–1.46)	0.71 (0.23–2.23) [a]

The hazard ratios (HR) and 95% confidence interval (CI), obtained using Cox Proportional Hazard models, are shown per standard deviation (SD) for the dietary patterns and scores. The basic model was adjusted for sex, age at baseline and Rotterdam Study cohort. The covariate model was adjusted for all items in the basic model and additionally for body mass index (BMI), education, smoking behavior and energy intake. [a] Due to an overfit model this covariate model was adjusted for all items in the basic model and additionally BMI and energy intake, but not for education and smoking behavior.

Table S4: Sex-stratified analyses for association between dietary patterns and Parkinson's disease.

	No. of Incident Parkinson's Disease/No. of Participants in Cohort	Basic Model HR (95%CI)	Covariate Model HR (95% CI)
Dutch diet quality score			
Female	61/5439	1.14 (0.88–1.49)	1.05 (0.81–1.38)
Male	68/3975	0.82 (0.63–1.06)	0.80 (0.61–1.04)
Mediterranean diet score			
Female	61/5439	1.02 (0.76–1.38)	0.93 (0.69–1.25)
Male	68/3975	0.87 (0.69–1.10)	0.86 (0.67–1.10)
Prudent pattern			
Female	61/5439	0.97 (0.65–1.44)	0.78 (0.50–1.23)
Male	68/3975	0.81 (0.57–1.16)	0.83 (0.56–1.22)
Traditional Dutch pattern			
Female	61/5439	1.36 (1.01–1.84)	1.13 (0.73–1.74)
Male	68/3975	0.83 (0.64–1.07)	0.80 (0.57–1.13)
Unhealthy pattern			
Female	61/5439	0.85 (0.6–1.19)	0.94 (0.67–1.32)
Male	68/3975	1.08 (0.83–1.39)	1.13 (0.86–1.49)

The hazard ratios (HR) and 95% confidence interval (CI), obtained using Cox Proportional Hazard models, are shown per standard deviation (SD) increase for the dietary patterns and scores. The basic model was adjusted for sex, age at baseline and Rotterdam Study cohort. The covariate model was adjusted for all items in the basic model and additionally for body mass index (BMI), education, smoking behavior and energy intake.

Table S5: Associations between dietary patterns and risk of parkinsonism (n = 9414).

Dietary Pattern	No. of Incident Parkinsonism [a]	Basic Model HR (95% CI)	Covariate Model HR (95% CI)
Dutch diet quality score per SD	254	0.97 (0.85–1.10)	0.95 (0.83–1.08)
Tertiles			
Low (reference)	71	1	1
Medium	95	1.04 (0.76–1.42)	1.01 (0.74–1.39)
High	88	1.10 (0.80–1.53)	1.05 (0.75–1.46)
Mediterranean diet score per SD	254	0.88 (0.78–1.01)	0.86 (0.76–0.98)
Tertiles			
Low (reference)	89	1	1
Medium	102	1.06 (0.79–1.41)	1.03 (0.77–1,38)
High	63	0.71 (0.51–0.98)	0.67 (0.48–0.94)
Prudent pattern, per SD	254	0.79 (0.64–0.96)	0.76 (0.61–0.95)
Tertiles			
Low (reference)	115	1	1
Medium	93	0.89 (0.67–1.18)	0.88 (0.67–1.17)
High	46	0.75 (0.52–1.08)	0.74 (0.50–1.08)
Unhealthy pattern, per SD	254	1.00 (0.86–1.15)	1.05 (0.90–1.22)
Tertiles			
Low (reference)	80	1	1
Medium	102	1.26 (0.94–1.69)	1.32 (0.98–1.78)
High	72	1.07 (0.76–1.50)	1.17 (0.83–1.66)
Traditional Dutch pattern, per SD	254	0.99 (0.86–1.13)	0.99 (0.82–1.20)
Tertiles			
Low (reference)	63	1	1
Medium	96	1.24 (0.89–1.71)	1.26 (0.90–1.77)
High	95	1.10 (0.78–1.55)	1.15 (0.76–1.73)

The hazard ratios (HR) and 95% confidence interval (CI), obtained using Cox Proportional Hazard models, are per standard deviation (SD) increase for the dietary patterns and scores. The basic model was adjusted for sex, age at baseline and Rotterdam Study cohort. The covariate model was adjusted for all items in the basic model and additionally for body mass index (BMI), education, smoking behavior and energy intake. [a] Parkinsonism includes: PD, drug-induced parkinsonism, vascular parkinsonism, Lewy Body disease, parkinsonism with dementia other than Lewy Body disease, multi system atrophy, progressive supranuclear palsy, corticobasal degeneration or parkinsonism resulting from a tumor.
